# Deformation Behavior of an Extruded 7075 Aluminum Alloy at Elevated Temperatures

**DOI:** 10.3390/ma17051210

**Published:** 2024-03-06

**Authors:** Tuo Ye, Erli Xia, Sawei Qiu, Jie Liu, Huanyu Yue, Jian Tang, Yuanzhi Wu

**Affiliations:** 1 School of Intelligent Manufacturing and Mechanical Engineering, Hunan Institute of Technology, Hengyang 421002, China; 2017001002@hnit.edu.cn (T.Y.); 2022001005@hnit.edu.cn (S.Q.); 2 Research Institute of Automobile Parts Technology, Hunan Institute of Technology, Hengyang 421002, China; 21010240132@xs.hnit.edu.cn (J.L.); 21010240108@xs.hnit.edu.cn (H.Y.); 21010240133@xs.hnit.edu.cn (J.T.)

**Keywords:** 7075 alloy, hot deformation, mechanical properties, microstructure, texture

## Abstract

Hot compression tests were conducted to explore the deformation behavior of an extruded 7075 aluminum alloy bar at elevated temperatures. Specimens with 0°, 45°, and 90° angles along the extrusion direction were prepared. The compression temperatures were 300 and 400 °C, and the strain rates ranged from 0.001 to 0.1 s^−1^. The corresponding microstructures were characterized via OM and TEM, and the macroscopic texture was tested using XRD. The results indicated that the strength of the 7075 alloy decreases with higher compression temperatures and is in a proportional relationship with respect to the strain rate. During high-temperature compression, it is easier to stimulate atomic diffusion in the matrix, which can improve thermal activation abilities and facilitate dynamic recovery and dynamic recrystallization. In addition, the coarsening of precipitates also contributed to dynamic softening. When compressed at 300 °C, the stress levels of the 0° specimens ranked first, and those for the 45° specimens were the lowest. When compressed at 400 °C, the flow stresses of the specimens along three directions were comparable. The anisotropic mechanical behavior can be explained by the fiber grains and brass {011} <211> texture component. However, higher temperature deformation leads to recrystallization, which can weaken the anisotropy of mechanical properties.

## 1. Introduction

Aluminum and its alloys are widely acknowledged for their outstanding performance: for example, desirable strength/weight ratio, excellent formability, low cost, exceptional corrosion resistance, and recyclability [1,2]. Therefore, aluminum and its alloy applications are increasingly adopted, and they are being used in aerospace and automotive fields, which can efficiently realize weight loss, reduce fuel consumption, and prevent environmental pollution [3,4]. Generally, 7075 aluminum alloys have high specific strength and prime fracture toughness; simultaneously, they have preferable resistance to stress corrosion cracking, which can guarantee their broad application [5]. In the aerospace field, they has been used to manufacture aircraft structures, fuselage shells, wings, propellers, and landing gear components [6,7]. In the automotive field, 7075 aluminum alloys are usually selected to produce automotive chassis, body structures, and engine components [8,9].

Moreover, 7075 aluminum alloys can be strengthened via heat treatment. By incorporating Zn, Mg, and Cu, which are the primary elements of precipitation, the service performance of 7075 aluminum alloys can be enhanced. However, this improvement may come at the cost of reduced workability and forming ability [10,11]. At ambient conditions, the plastic deformation ability of 7075 aluminum alloys is not entirely satisfactory, and achieving the required forming accuracy for the application is challenging [12,13]. Therefore, hot deformation processes, such as hot rolling, hot extrusion, and hot stamping, are commonly adopted to produce aluminum alloy components [14,15,16]. The exploration of deformation behavior and the related microstructure mechanism of the alloys at higher temperature conditions is of great importance for examining workability characteristics, which are beneficial for guaranteeing the precise control of the aluminum alloy component and improving their mechanical properties. In recent years, several research studies have focused on the deformation response of 7XXX-series alloys at various deformation conditions. Liu et al. [17] probed the isothermal compression response of the AA7085 alloy. The deformation temperature was between 250 °C and 450 °C, and the deformation strain rate was between 0.01 s^−1^ and 10 s^−1^; the findings suggest that the stress levels decreased with higher temperatures or lower deformation strain rates. Cai et al. [18] investigated the isothermal compression response of an Al-Zn-Mg-Cu alloy, and the experimental data indicated that the strength of the alloy decreased with increasing temperatures and retained a proportional relationship with the strain rate. The influence of thermal deformation conditions on mechanical performance was discussed, and the results were employed to optimize the hot processing of the alloy. Lei et al. [19] explored the isothermal uniaxial compress response of a 7XXX-series alloy in cast condition, and a strain-compensated Arrhenius-type model was adopted to describe the flow stress, which is of great importance in the forming process of alloys in component manufacturing. Guo et al. [20] selected 7050 alloys to examine the hot deformation response. The temperatures were between 150 °C and 450 °C, and the strain rates were between 0.1 s^−1^ and 1 s^−1^. The test results indicated that both temperatures and strain rates are decisive relative to the mechanical performance of the material, and a physical model was proposed to express the flow behavior, which takes the microstructure’s evolution into consideration, such as the evolution of dislocation, precipitates, and solutes, having the ability to provide a more accurate prediction in the processing of alloys and their components. In conclusion, a series of research studies on the mechanical responses of 7XXX-series alloys was conducted, and they focused on stress–strain curves, temperature sensitivity, strain rate sensitivity, constitutive equations, etc. However, reports about anisotropic mechanical properties and the effects of temperature and strain rates on anisotropic behavior are rarely found; therefore, it is worth exploring anisotropic deformation behaviors at different temperatures and strain rates.

It is worth noting that during the process of hot deformation, the microstructure of metal materials will experience tremendous change. On the one hand, the interaction and competition between hardening effects and softening effects are complicated and long-lasting. For an aluminum alloy, during plastic deformation processes at elevated temperatures, the recovery, recrystallization, grain refinement and coarsening, and evolution of precipitates (dynamic precipitation, dynamic growth, and dynamic dissolution) will occur, and they have substantial influence on the compressive behavior of the alloy. Ke [21] studied 7020 aluminum alloys and found that when they were deformed within 470 to 520 °C and 0.004 to 0.05 s^−1^, the alloys exhibited a typical dynamic recovery characteristic. When deformed within 460 to 520 °C and 0.001 to 0.004 s^−1^, partial recrystallization was observed. When deformed within 400 to 460 °C and 0.001 to 0.1 s^−1^, the phenomenon of localization deformation was observed. Wu [22] explored the compression response and microstructure mechanism of a new Al-Zn-Mg-Cu alloy from 300 to 420 °C and 0.01 to 10 s^−1^. The characterization results demonstrated that the grain size increases with increasing deformation temperatures; meanwhile, the fraction of DRX increases with increasing temperatures. In addition, the grain size becomes coarser with decreasing strain rates. In contrast, the fraction of DRX first increases and then decreases with increasing temperatures. Sun et al. [23] conducted a thermal compression experiment on as-extruded 7075 aluminum alloy, and they found that higher temperatures and lower strain rate deformation conditions are more likely to result in the occurrence of dynamic recrystallization and grain coarsening, which lead to a further decrease in strength and hardness. Luo et al. [24] explored the compression response of a 7XXX-series alloy. The temperature was between 320 and 440 °C, and the strain rate was between 0.01 and 10 s^−1^. The phenomena of precipitate coarsening and dynamic precipitate dissolution were observed, which lead to a decline in strength. On the other hand, during hot plastic deformation, the orientation of grains will occur and take evolution during hot processes. Guo [25] explored the anisotropic response of a 7XXX-series alloy, and they found that strong textures result in decisive effects relative to anisotropic mechanical behaviors. Li [26] investigated the anisotropy of an Al-Zn-Mg-Cu alloy, and they found that {110} <112> brass texture formed during the rolling process contributed to the anisotropic response. Srinivasan et al. [27] examined the equibiaxial tension of an Al-Zn-Mg-Cu alloy. They concluded that the initial brass texture weakens during equibiaxial deformation. On the whole, a substantial number of previous research studies have revealed the grain, precipitate, and dislocation evolution of 7XXX-series alloys at elevated temperatures, and discussions of the deformation mechanism based on the observation of microstructure evolution were carried out. However, the microstructure mechanism of anisotropic deformation behaviors at elevated temperatures requires further and more comprehensive probing.

In total, the evolution of microstructures, such as grains, dislocations, precipitates, and textures, has a significant impact on the formability and service performance of materials. Hot deformation parameters can determine the microstructure of materials. In this case, 7075 aluminum alloy components are typically used at from a room temperature up to 300 °C, and they are formed at temperatures ranging from 300 to 400 °C. As a result, exploring compression responses and the corresponding microstructure mechanism at elevated temperatures is needed. A more comprehensive and in-depth study of deformation regulation and deformation mechanisms is important for product creation, process adjustment, forming control, and property improvement in the manufacturing industry.

## 2. Experimental Section

### 2.1. Materials

**Figure 1:**
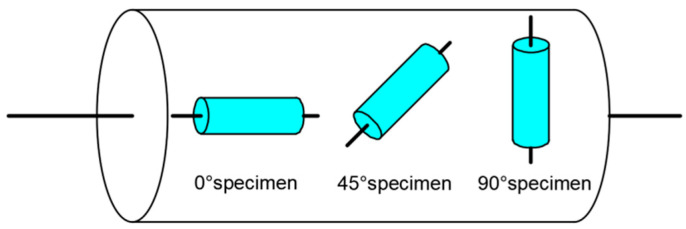
The diagram of the sample location in the original 7075 aluminum alloy bar.

In this study, an Al-5.8Zn-2.3Mg-1.5Cu alloy was chosen as the test material. The studied alloy comprised an extrusion bar with a diameter of 35 mm. As displayed in Figure 1, the samples for the hot compression test were cut from the bar at 0°, 45°, or 90° along the extrusion direction (ED). The height and diameter of the cylindrical samples were 12 mm and 8 mm, respectively.

### 2.2. High-Temperature Compression Test

**Figure 2:**
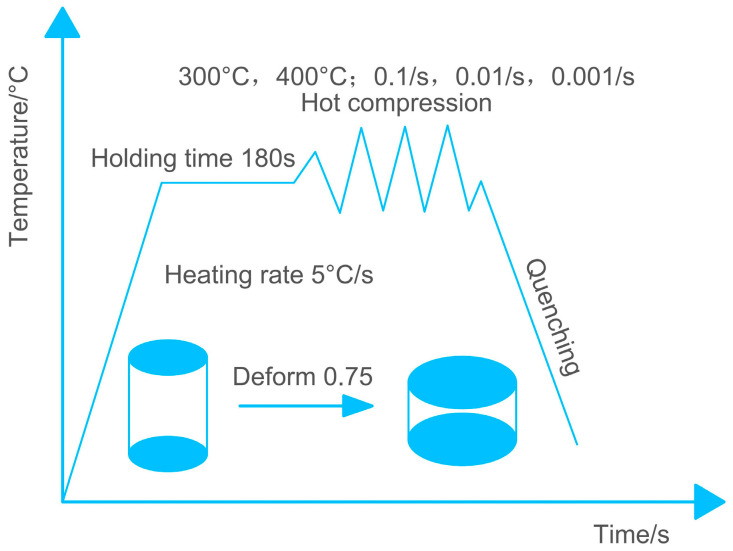
Diagram of the hot compression processes and parameters.

The compression test at elevated temperatures was conducted to explore the deformation response of the alloy. Detailed compression processes are displayed in Figure 2. The experiments were conducted using a Gleeble-3800 thermal simulation machine (DSI, St. Paul, MN, USA). The heating rate was 5 °C/s, compression temperatures were 300 and 400 °C, and strain rates were 0.001 s^−1^, 0.01 s^−1^, and 0.1 s^−1^. Once the target temperature was achieved, the test samples were held for 180 s. True strain was selected as 0.75, and the cooling method used after compression was water quenching. For each test condition, three valid experiments were conducted.

### 2.3. Characterization

**Figure 3:**
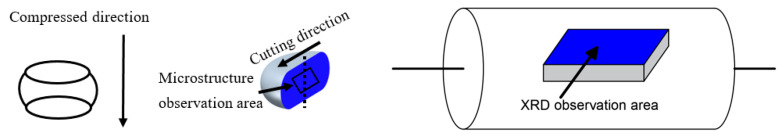
Microstructure and XRD observation area of the sample.

**Figure 4:**
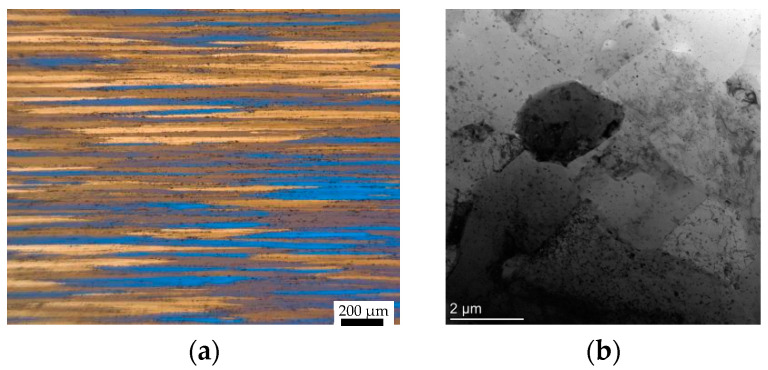
The initial microstructure of 7075 aluminum alloy: (**a**) optical microscope (OM) observation and (**b**) transmission electron microscope (TEM) observation.

The microstructure and XRD observation area of the sample are shown in Figure 3. Microstructure characterization surfaces were cut from the deformed samples. A mirror surface can be obtained via grinding and polishing. Subsequently, the surfaces were electrolytically etched to obtain the microstructure’s characteristics. The etching solution comprised water and HBF_4_ (5% by volume) (Runfeng Petrochemical Co., Ltd., Nantong, China). The etching parameters were set at 20 V, and etching was carried out from 2 to 4 min. Moreover, microstructure characterization was carried out using an optical microscope. For TEM characterization, slices with a thickness of 0.5 mm were machined via wire electrical discharge. Subsequently, the slice was reduced to 70 μm. Then, the slice was thinned and perforated via double spraying. The microstructures were characterized using Talos F200X (FEI, Hillsboro, OR, USA). An X-ray diffractometer (BRUKER, Billerica, MA, USA) was used to test and analyze the texture. The observation surface was ground and polished, and the pole figures of (111), (200), (220), and (311) were tested. The initial microstructures of the 7075 aluminum alloy are displayed in Figure 4. The extruded 7075 aluminum alloy bar consisted of elongated grains, and a certain number of dislocations and precipitates were observed.

## 3. Experimental Results

**Figure 5:**
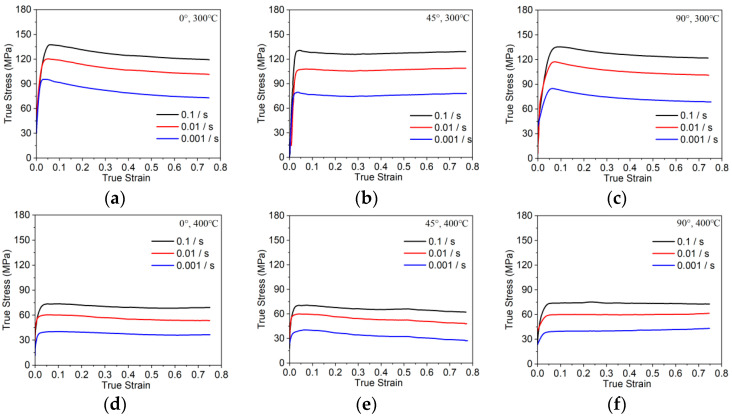
True stress–strain curves of 7075 alloy bar with different compression conditions: (**a**) 0°, 300 °C; (**b**) 45°, 300 °C; (**c**) 90°, 300 °C; (**d**) 0°, 400 °C; (**e**) 45°, 400 °C; (**f**) 90°, 400 °C.

Figure 5 shows the true stress–true strain curves of extruded 7075 alloys after testing at different compression conditions. For the 7075 alloys, during the initial stage of compression, their true stress underwent rapid growth. For example, when deformed at 300 °C, the true stress of the 0° specimen swiftly increased to 137 MPa, 119 MPa, and 95 MPa at strain rates of 0.1, 0.01, and 0.001 s^−1^, respectively. Once the yield stress is obtained, the stress of the alloy increases at a moderate speed. In general, microstructure evolution during hot compression comprises intense and sustained competition between hardening and softening effects. Hardening plays a dominant role, and it is caused by a series of dislocation evolutions, namely rapid multiplication, extensive accumulation, and fierce interaction [28]. At this stage, the effect of dynamic softening is not obvious. In essence, dislocation annihilation resulted from dynamic softening, and it cannot offset the dislocation generation caused by work hardening. As the deformation continues, the intensity of work hardening declines. Dynamic softening occurs, which facilitates the annihilation of dislocation, slows down the dislocation interaction, and results in a decrease in dislocation density [29]. As a result, the strength effect of work hardening deteriorates. However, work hardening is not capable of offsetting the effects of dynamic softening during hot compression. As a result, the stress–strain curves exhibit a descending trend until the target strain is obtained.

As shown in Figure 5, both the compression temperature and strain rate have direct impacts on the stress level of the extruded 7075 aluminum alloy. To summarize this phenomenon, at a constant strain rate, the stress levels of the 7075 alloy decrease with increasing compression temperatures. We use the 0° samples as an example. As shown in Table 1, when the deformed parameters are 0.001 s^−1^/300 °C and 0.001 s^−1^/400 °C, the yield stresses are 95 MPa and 38 MPa, respectively. Meanwhile, at a constant compression temperature, the stress levels of the 7075 alloy exhibit a downward trend with decreasing strain rates. When deformed at 400 °C, the yield stresses are 73 MPa, 60 MPa, and 38 MPa at 0.1 s^−1^, 0.01 s^−1^, and 0.001 s^−1^, respectively. This hot compression behavior could be expressed by analyzing and discussing the interactions among the temperature, strain rate, and softening effect. It is commonly acknowledged that dynamic softening is a thermally activated process [30]. As the compression temperature increases, the mobility of dislocations and boundaries is improved. Consequently, the density of the dislocation and the resistance against the motion of dislocation decrease considerably. Therefore, the effect of dynamic softening is significantly strengthened. Meanwhile, the generation speed of the dislocation decreases with lower strain rates. As a result, the barriers that can limit the motion of dislocation are reduced. In addition, a lower strain rate can provide more time to accumulate activation energies and promote a softening effect. Hence, the stress level of the 7075 alloy obviously decreases at a relatively lower strain rate.

The yield stresses of the materials manufactured along different directions of the bar are shown in Figure 5. Overall, when compressed at 300 °C, the yield stresses of the 0° samples always ranked the highest, the yield stresses of the 90° samples were comparable to those of the 0° samples, and the stress values of the 45° samples ranked at the bottom. For instance, when the samples were deformed relative to the conditions of 300 °C and 0.1 s^−1^, the corresponding yield stress values were 137 MPa, 129 MPa, and 136 MPa for the 0°, 45°, and 90° samples, respectively. Similar trends of mechanical anisotropy have been observed in previous studies, and it was reported that fibrous grains and deformation texture are responsible for this form of regulation [31,32]. During plastic deformations, the grain boundary would hinder the movement of dislocations, and elongated grains result due to the different densities of the grain boundary along three loading directions. Therefore, the diversity of grain boundary densities contributed to mechanical anisotropy. However, as compression temperatures increased to 400 °C, anisotropic property phenomena disappeared. The yield stress of the samples along three directions was demonstrated to be comparable. For instance, when compressed at the condition of 400 °C and 0.001 s^−1^, the yield stresses of samples along the three directions are about 38 MPa. Higher compression temperatures can produce increased energy, which can improve processes such as dynamic recovery and dynamic recrystallization [33]. The density of dislocation could also rapidly decrease, and, simultaneously, slip activation occurs easily as the slip is unrestrained along different directions. Meanwhile, the original deformed textures that formed during the extrusion process begin to transform into recrystallized textures [34], which can efficiently weaken the anisotropy of the alloy.

## 4. Discussion

### 4.1. Dynamic Softening Behavior

**Figure 6:**
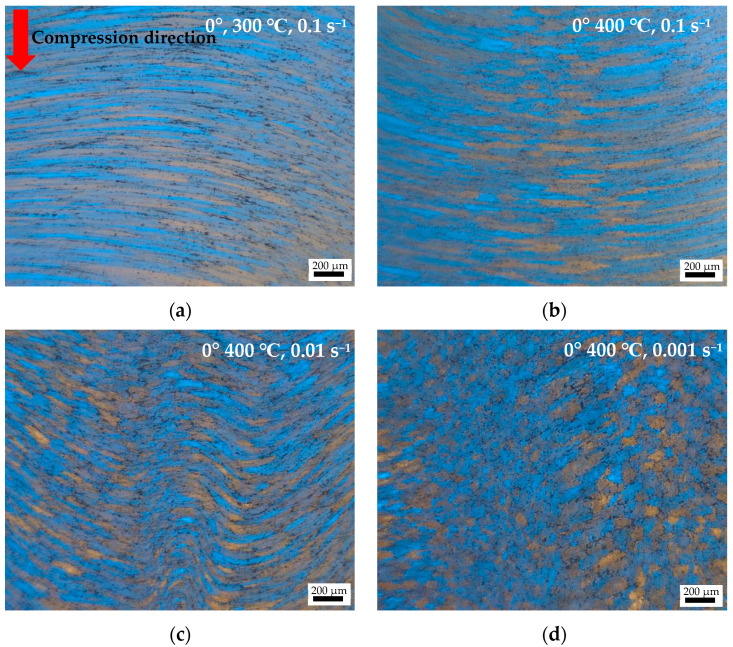
OM of 7075 alloys compressed using different conditions: (**a**) 0°, 300 °C, 0.1 s^−1^; (**b**) 0°, 400 °C, 0.1 s^−1^; (**c**) 0°, 400 °C, 0.01 s^−1^; (**d**) 0°, 400 °C, 0.001 s^−1^.

During hot compression, the original grains are twisted along the shear direction, and no cracks or voids are present. As shown in Figure 6, when compressed at 300 °C and 0.1 s^−1^, the grains underwent deformations due to external forces. However, during the deformation process, the stress at different parts of the grains was uneven, and the main stress area of the grains underwent twisted deformations due to the shear stress concentration. This demonstrates that restored energy was not sufficient for activating the DRX, and dynamic recovery was still dominant, with the average width of the grain being 32–38 μm. Compared with compression at 300 °C, the fiber structure exhibited a certain degree of growth in the transverse direction after compression at conditions of 400 °C and 0.1 s^−1^, with an average width of the grain being 40–50 μm. As shown in Figure 6b, fiber grains with larger cross-sections are observed. The grains underwent torsional deformation under shear force action. As the strain rate decreased to 0.01 s^−1^, the fiber grains after deformation underwent significant disintegration, and the fiber’s length was significantly shorter, with the average length of the grain being 190–200 μm. In addition, recrystallized grains have appeared in some areas. When the strain rate decreased to 0.001 s^−1^ at a deformation temperature of 400 °C, the deformed fiber structure underwent more significant disintegration, and the original slender fiber structure evolved into multiple parallel recrystallized grains distributed along the fiber’s direction. The alloy’s grain size significantly decreased, and some grains exhibited equiaxed recrystallization characteristics, with average length of the grain being 85–100 μm. Higher temperatures and lower strain rate deformations are beneficial for recovery and recrystallization processes, resulting in a decrease in alloy strength.

**Figure 7:**
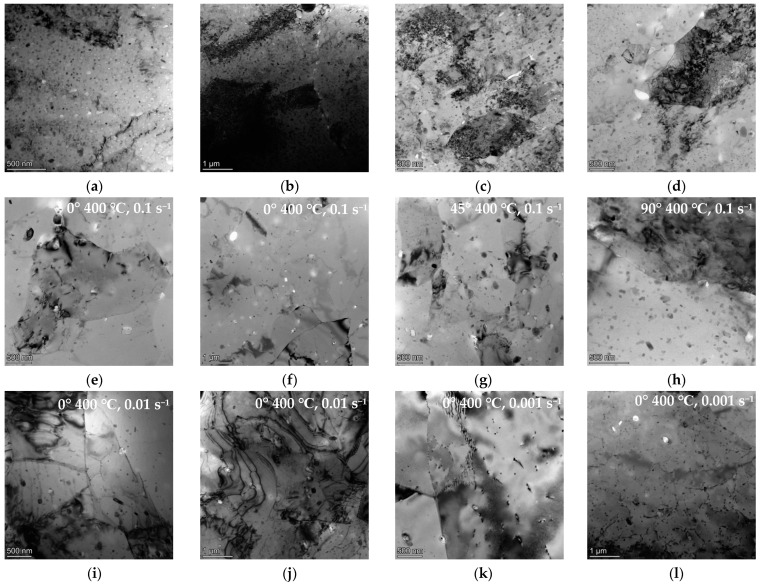
TEM images of 7075 alloy samples after deformation under different conditions: (**a**) 0°, 300 °C, 0.1 s^−1^; (**b**) 0°, 300 °C, 0.1 s^−1^; (**c**) 45°, 300 °C, 0.1 s^−1^; (**d**) 90°, 300 °C, 0.1 s^−1^; (**e**) 0°, 400 °C, 0.1 s^−1^; (**f**) 0°, 400 °C, 0.1 s^−1^; (**g**) 45°, 400 °C, 0.1 s^−1^; (**h**) 90°, 400 °C, 0.1 s^−1^; (**i**) 90°, 400 °C, 0.01 s^−1^; (**j**) 0°, 400 °C, 0.01 s^−1^; (**k**) 0°, 400 °C, 0.001 s^−1^; (**l**) 0°, 400 °C, 0.001 s^−1^.

Figure 7 shows the TEM characterization results of 7075 alloys after hot deformation. After compression at 300 °C and 0.1 s^−1^, the microstructure of the samples in all three directions is consistent. The matrix mainly comprises deformed microstructures, with a certain number of small-sized precipitates that are spread in the aluminum alloy. When compression deformation occurs, the precipitates would effectively hinder the motion of dislocations. Therefore, a substantial number of dislocation lines are tangled together, with obvious dislocation accumulation. This indicates that the alloy undergoes dynamic recovery under compression at this temperature. When the temperature increases to 400 °C under 0.1 s^−1^ conditions, the microscopic characteristics of the samples in all three directions are basically consistent, with obvious small subgrains appearing. As the temperature increases, dislocations disappear through the slip and are rearranged, resulting in a gradual decrease in dislocation density inside the subgrain, clearer subgrain boundaries, and larger sizes. Meanwhile, it was also observed that the precipitates in the matrix exhibited coarsening phenomena. As shown in Figure 7i–l, as the strain rate decreases, the cross slip and climb of dislocations during compression under conditions of 400 °C and 0.01 s^−1^ as well as 400 °C and 0.001 s^−1^ result in the mutual annihilation of dissimilar dislocations, and polygonization processes also occur. As compression occurs at a higher strain rate, the pile-up of the dislocation is obvious, and the density of the dislocation increases significantly, which is beneficial for dynamic recrystallization. Therefore, higher strain rates result in lower dynamic recrystallization temperatures. However, high-density dislocations formed by deformations rapidly increase the energy in the matrix. The hot and deformed metal overcomes the pinning effect of deformation textures in order to modify this unstable state. Numerous studies have shown that 7075 alloys have high stacking fault energy and relatively weak diffusion abilities. Therefore, dynamic recovery mainly occurs during high-temperature deformation. The polygonization process is achieved via the climb and cross-slip movements of dislocations, resulting in the formation of subgrain structures. Therefore, dynamic recovery is the leading softening effect during hot compressions. The flow stress during the high-temperature compression process of extruded 7075 alloys is mainly determined by the interaction effect between work hardening and dynamic recovery. From a microscopic perspective, it essentially comprises the process of dislocation generation, movement, and annihilation.

### 4.2. Anisotropic Deforming Behavior

**Figure 8:**
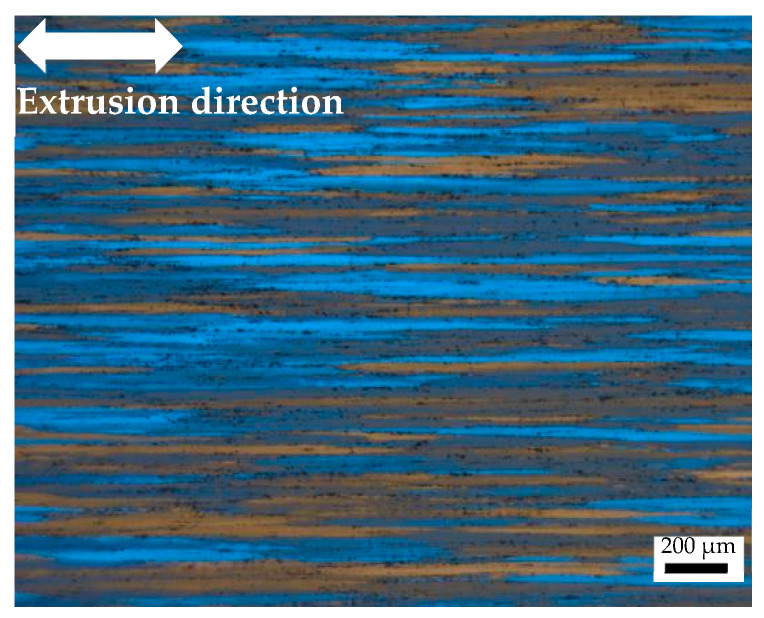
Original grain structure of the extruded 7075 aluminum alloy.

During deformation, many factors result in alloys exhibiting anisotropic mechanical properties regarding their grain shape, texture, particle distribution, and chemical composition distribution. In this work, grain shape and texture are mainly discussed. Figure 8 displays the grain structure of the extruded 7075 aluminum alloy. Most grains are elongated towards the extrusion direction. The grain boundaries of the 7075 alloy are clear. The width of the grains is within the range of 30–40 µm. Preliminary studies prove that elongated grains cause alloys to exhibit anisotropic mechanical properties [35]. Yang [36] conducted a hot compression test of an Al-Zn-Mg-Cu alloy at a temperature range from 320 to 340 °C. The alloy with elongated grains exhibited anisotropic compression responses. The experimental data imply that the specimens along the extrusion direction exhibit the maximum strength; meanwhile, the strength of the 90° specimen is the lowest. Qiu [31] investigated the response of a 6082 alloy processed via extrusion, and it was reported that the specimens along the extrusion direction have the highest stress level. This can be attributed to the elongated grains that formed during extrusion.

Generally speaking, grain boundaries can hinder the movement of dislocations during compression. Indeed, for samples along different directions, elongated grains can result in different grain boundary densities. During the process of compression, dislocation accumulation will occur along the grain boundary, which will trigger lattice distortion; consequently, resistance relative to the deformation will be enhanced. In other words, different boundary densities have various strengthening effects. Therefore, different anisotropic mechanical behaviors can be explained [37].

Adopting the Schmid factor and orientation distribution functions (ODFs) to explain anisotropy is meaningful [38,39]. The texture type and intensity can be achieved using ODFs, and then the Schmid factor for each texture component of different direction specimens can be calculated based on the crystal model. The specimen with a higher Schmid factor value exhibits lower stress. Therefore, the effect of orientation on the mechanical properties of an alloy can be explained using Schmid factors and orientation distribution functions (ODFs):

(1) $$\tau =\sigma_{y}=\frac{F}{A}\cos\varphi \cos\lambda$$

(2) M=cosφcosλ

where τ is the critical shear stress, *A* is the loading section, φ is the angle between the loading axis and the normal relative to the slip plane, λ is the angle between the loading axis relative to the slip direction, $\sigma_{y}$ is the yield stress, and *M* is the Schmid factor. Yang et al. [36] investigated the anisotropic deformation behavior of a 7XXX-series alloy processed via extrusion. The corresponding SF was calculated, and the samples along the extrusion exhibited the minimum SF. Therefore, the strength of samples along the extrusion direction was the highest. Ye et al. [40] selected a 6063 alloy bar to investigate the mechanism of anisotropy. The SFs for the samples along different directions were determined, and the results showed that the SF of the 45° sample was at the maximum; correspondingly, the strengths of the 45° sample were the lowest.

**Figure 9:**
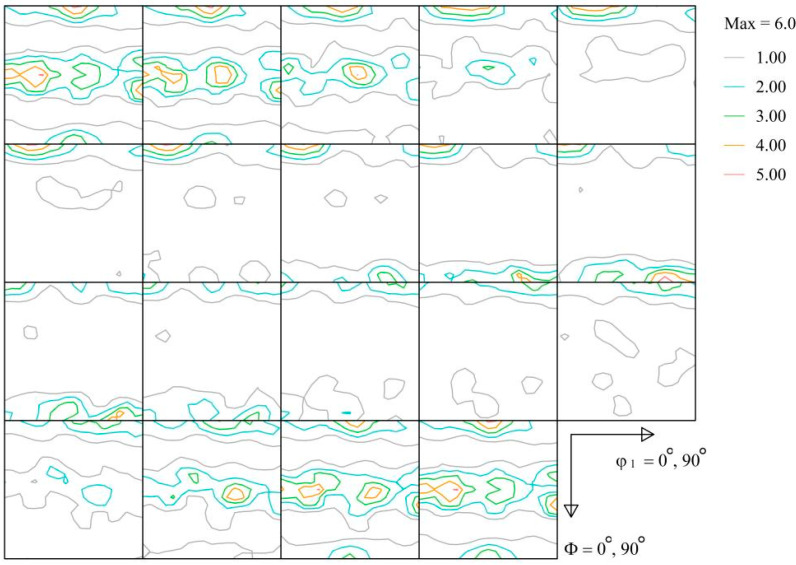
XRD-derived ODF sections of macrotextures measured in the extruded 7075 aluminum alloy bar.

Figure 9 shows the ODF sections for the 7075 aluminum alloy bar. The 7075 aluminum alloy bar exhibits significant orientations. The dominant component is {011} <211> brass texture, which is a classic deformation texture. Prior studies proved that deformation textures could cause anisotropic mechanical behavior. On the contrary, recrystallization texture may deteriorate or eliminate anisotropy. When compressed at a temperature of 300 °C, energy is insufficient with respect to triggering dynamic recrystallization; therefore, the {011} <211> brass component contributed to the anisotropic behavior. However, when compressed at a temperature of 400 °C, dynamic recrystallization is achieved. The {011} <211> brass component may deteriorate or disappear, and accordingly, the studied alloy exhibits isotropic mechanical behavior.

## 5. Conclusions

(1) The stress level of the 7075 alloy bar decreased with an increase in compression temperature and a decrease in strain rate. In addition, the extruded 7075 aluminum alloy exhibited anisotropic mechanical behavior at a deformation temperature of 300 °C. However, no evident anisotropy was observed at a deformation temperature of 400 °C.
(2) During compression at 300 °C, dynamic recovery occurred. The dislocation density decreased at higher compression temperatures; simultaneously, with a decrease in strain rate, the density of dislocations exhibited a decreasing trend. Moreover, when compressed at 400 °C, a certain number of equiaxed grains were found, which demonstrated the occurrence of dynamic recrystallization. At the same time, the precipitates became coarser during compression. The softening effect, including dynamic recovery, dynamic recrystallization, and precipitate coarsening, contributed to a decrease in strength.
(3) When compressed at 300 °C, the extruded 7075 alloy bar exhibited anisotropic strength. This can be explained by the elongated grains and the {011} <211> brass component. When compressed at 400 °C, no evident anisotropic mechanical properties were observed. Higher compression temperatures can produce more energy in order to activate and promote slipping. In addition, dynamic recrystallization results in a change in grain shape and the evolution of the orientation.

## Figures and Tables

**Table 1:** Max stress of 7075 aluminum alloy with different conditions.

Strain Rate/s^−1^	Temperature/°C	Direction			Temperature/°C	Direction		
		0°	45°	90°		0°	45°	90°
0.1	300	137	129	136	400	73	72	72
0.01		119	106	116		60	59	59
0.001		95	79	84		38	38	38

## Data Availability

Data are contained within this article.

## References

[1] Berlanga-Labari C., Biezma-Moraleda M.V., Rivero P.J. (2020). Corrosion of cast aluminum alloys. Metals.

[2] Noga P., Skrzekut T., Wędrychowicz M., Węglowski M.S., Węglowska A. (2023). Research of friction stir welding (FSW) and electron beam welding (EBW) process for 6082-T6 aluminum alloy. Materials.

[3] Bhatta L., Pesin A., Zhilyaev A.P., Tandon P., Kong C., Yu H. (2020). Recent development of superplasticity in aluminum alloys. Metals.

[4] Vatansever F., Erturk A.T., Karabay S. (2019). Improving mechanical properties of AlSi10Mg aluminum alloy using ultrasonic melt treatment combined with T6 heat treatment. Kov. Mater..

[5] Bamberg P., Gintrowski G., Liang Z., Schiebahn A., Reisgen U., Precoma N., Geffers C. (2021). Development of a new approach to resistance spot weld AW-7075 aluminum alloys for structural applications: An experimental study–Part 1. J. Mater. Res. Technol..

[6] Younis H.B., Kamal K., Sheikh M.F., Hamza A. (2022). Prediction of fatigue crack growth rate in aircraft aluminum alloys using optimized neural networks. Theor. Appl. Fract. Mec..

[7] Baek M.S., Euh K., Lee K.A. (2020). Microstructure, tensile and fatigue properties of high strength Al 7075 alloy manufactured via twin-roll strip casting. J. Mater. Res. Technol..

[8] Choi Y., Lee J., Panicker S.S., Jin H.K., Panda S.K., Lee M.G. (2020). Mechanical properties, springback, and formability of W-temper and peak aged 7075 aluminum alloy sheets: Experiments and modeling. Int. J. Mech. Sci..

[9] Hattori C.S., Almeida G.F.C., Gonçalves R.L.P., Santos R.G., Souza R.C., da Silva W.C., Couto A.A. (2021). Microstructure and fatigue properties of extruded aluminum alloys 7046 and 7108 for automotive applications. J. Mater. Res. Technol..

[10] Whalen S., Olszta M., Reza-E-Rabby M., Roosendaal T., Wang T., Herling D., Overman N. (2021). High speed manufacturing of aluminum alloy 7075 tubing by Shear Assisted Processing and Extrusion (ShAPE). J. Manuf. Process..

[11] Otani Y., Sasaki S. (2020). Effects of the addition of silicon to 7075 aluminum alloy on microstructure, mechanical properties, and selective laser melting processability. Mat. Sci. Eng. A.

[12] Peterson L.A., Horstemeyer M.F., Lacy T.E., Moser R.D. (2020). Experimental characterization and constitutive modeling of an aluminum 7085-T711 alloy under large deformations at varying strain rates, stress states, and temperatures. Mech. Mater..

[13] Shiraiwa T., Briffod F., Enoki M. (2023). Prediction of fatigue crack initiation of 7075 aluminum alloy by crystal plasticity simulation. Materials.

[14] Alewi D., Murdoch H., Magagnosc D., Lemmen K., Karaca H., Rottmann P. (2024). Depth-dependent microstructure and mechanical properties of hot rolled AA7075. Mat. Sci. Eng. A.

[15] Noga P., Piotrowicz A., Skrzekut T., Zwoliński A., Strzępek P. (2021). Effect of various forms of aluminum 6082 on the mechanical properties, microstructure and surface modification of the profile after extrusion process. Materials.

[16] Scharifi E., Nietsch J.A., Quadfasel A., Weidig U., Steinhoff K. (2022). Effect of thermo-mechanically activated precipitation on the hot deformation behavior of high strength aluminum alloy AA7075. Metals.

[17] Liu W., Zhao H., Li D., Zhang Z., Huang G., Liu Q. (2014). Hot deformation behavior of AA7085 aluminum alloy during isothermal compression at elevated temperature. Mat. Sci. Eng. A.

[18] Cai J., Chen L., Yang J., Wang W., Ding B., Yang Q., Wang K. (2022). Hot Deformation behavior and microstructure evolution of high-strength Al-Zn-Mg-Cu alloy. Metals.

[19] Lei C., Wang Q., Ebrahimi M., Li D., Tang H., Zhang N., Cai H. (2023). Hot Deformation behavior and processing maps of an as-cast Al-5Mg-3Zn-1Cu (wt%) alloy. Materials.

[20] Guo R., Liang D., Qin G. (2023). The flow stress behavior and physical-based constitutive model for as-quenched Al-Zn-Mg-Cu alloy. Materials.

[21] Ke B., Ye L., Tang J., Zhang Y., Liu S., Lin H., Liu X. (2020). Hot deformation behavior and 3D processing maps of AA7020 aluminum alloy. J. Alloy. Compd..

[22] Wu S., Zhu B., Jiang W., Qiu H., Guo Y. (2022). Hot deformation behavior and microstructure evolution of a novel Al-Zn-Mg-Li-Cu alloy. Materials.

[23] Sun Z.C., Zheng L.S., Yang H. (2014). Softening mechanism and microstructure evolution of as-extruded 7075 aluminum alloy during hot deformation. Mater. Charact..

[24] Luo L., Liu Z., Bai S., Zhao J., Zeng D., Wang J., Hu Y. (2020). Hot deformation behavior considering strain effects and recrystallization mechanism of an Al-Zn-Mg-Cu Alloy. Materials.

[25] Guo Y., Zhang M., Wang Z., Wang S., Liu C., Qian L., Zhao H. (2021). Effects of cold temperatures, strain rates and anisotropy on the mechanical behavior and fracture morphology of an Al–Zn–Mg–Cu alloy. Mat. Sci. Eng. A.

[26] Li Y., Xu G., Liu S., Wang B., Peng X. (2021). Study on anisotropy of Al-Zn-Mg-Sc-Zr alloy sheet. Mater. Charact..

[27] Srinivasan N., Velmurugan R., Bhaskar L.K., Singh S.K., Pant B., Kumar R. (2021). The role of brass texture on the deformation response of 7075-T651 aluminum alloy under equi-biaxial tension. Mat. Sci. Eng. A.

[28] Raja N., Kumar A., Patel S.K. (2023). Hot deformation and microstructural evolution of ultrasonically fabricated as-cast Al-7.3 Zn-2.2 Mg-2 Cu alloy. Mater. Charact..

[29] Österreicher J.A., Tunes M.A., Grabner F., Arnoldt A., Kremmer T., Pogatscher S., Schlögl C.M. (2020). Warm-forming of pre-aged Al-Zn-Mg-Cu alloy sheet. Mater. Design.

[30] Dubey R., Jayaganthan R., Ruan D., Gupta N.K., Jones N., Velmurugan R. (2023). Energy absorption and dynamic behaviour of 6xxx series aluminium alloys: A review. Int. J. Impact Eng..

[31] Qiu S., Xia E., Liu L., Ye T., Liu J., Tang J., Wu Y. (2023). Tensile behavior and microstructure evolution of an extruded 6082 aluminum alloy sheet at high temperatures. Metals.

[32] Frodal B.H., Thomesen S., Børvik T., Hopperstad O.S. (2022). On fracture anisotropy in textured aluminium alloys. Int. J. Solids Struct..

[33] Maizza G., Pero R., Richetta M., Montanari R. (2018). Continuous dynamic recrystallization (CDRX) model for aluminum alloys. J. Mater. Sci..

[34] Attarilar S., Ebrahimi M., Hsieh T.H., Uan J.Y., Göde C. (2021). An insight into the vibration-assisted rolling of AA5052 aluminum alloy: Tensile strength, deformation microstructure, and texture evolution. Mat. Sci. Eng. A.

[35] Thomesen S., Hopperstad O.S., Børvik T. (2021). Anisotropic plasticity and fracture of three 6000-series aluminum alloys. Metals.

[36] Yang Y., Xie Z., Zhang Z., Li X., Wang Q., Zhang Y. (2014). Processing maps for hot deformation of the extruded 7075 aluminum alloy bar: Anisotropy of hot workability. Mat. Sci. Eng. A.

[37] Lim H., Lee M.G., Kim J.H., Adams B.L., Wagoner R.H. (2011). Simulation of polycrystal deformation with grain and grain boundary effects. Int. J. Plast..

[38] Raabe D., Roters F. (2004). Using texture components in crystal plasticity finite element simulations. Int. J. Plast..

[39] Bois-Brochu A., Blais C., Goma F.A.T., Larouche D. (2016). Modelling of anisotropy for Al-Li 2099 T83 extrusions and effect of precipitate density. Mat. Sci. Eng. A.

[40] Ye T., Li L.X., Liu X., Liu W.H., Guo P.C., Tang X. (2016). Anisotropic deformation behavior of as-extruded 6063-T4 alloy under dynamic impact loading. Mat. Sci. Eng. A.

